# Retinal functional and structural changes in patients with Parkinson’s disease

**DOI:** 10.1186/s12883-023-03373-6

**Published:** 2023-09-18

**Authors:** Rehab Elanwar, Hatem Al Masry, Amna Ibrahim, Mona Hussein, Sahar Ibrahim, Mohammed M. Masoud

**Affiliations:** 1 Neuro Diagnostic Research Center, Beni-Suef University, Beni-Suef, Egypt; 2 Department of Neurology, Beni-Suef University, Salah Salem Street, Beni-Suef, Egypt; 3 Department of Ophthalmology, Beni-Suef University, Beni-Suef, 62511 Egypt

**Keywords:** Parkinson’s disease, Optical coherence tomography, Full field electroretinogram, Retinal nerve fiber layer thickness, Macular ganglion cell complex

## Abstract

**Background:**

Visual dysfunction have been well reported as one of the non-motor symptoms in Parkinson’s disease (PD). The aim of this study was to evaluate the functional and structural changes in the retina in patients with PD, and to correlate these changes with disease duration and motor dysfunction.

**Methods:**

For this case–control study, we recruited patients fulfilling the diagnostic criteria for idiopathic PD according to British Brain Bank criteria, aged between 50 and 80 years. Age- and sex-matched healthy controls aged between 50 and 80 years were also recruited. Motor function for PD patients was assessed using Modified Hoehn and Yahr staging scale (H & Y staging) and Unified Parkinson’s Disease Rating Scale (UPDRS). Optical Coherence Tomography (OCT) and full field electroretinogram (ff-ERG) were done to all participants.

**Results:**

Data from 50 patients and 50 healthy controls were included in the analysis. Patients with idiopathic Parkinson's had significantly reduced peripapillary retinal nerve fiber layer (RNFL) thickness and macular ganglion cell complex (GCC) thickness compared to healthy controls (*P*-value < 0.05 in all parameters). They also had significantly delayed latency and reduced amplitude in both dark-adapted rods and the light-adapted cone for both a & b waves compared to healthy controls (*P*-value < 0.001 in all parameters). There were statistically significant negative correlations between disease duration, and left superior, right inferior and right & left average RNFL thickness [(r) coef. = -0.327, -0.301, -0.275, and -0.285 respectively]. UPDRS total score was negatively correlated with the amplitude of light-adapted of both RT and LT a & b wave and with dark-adapted RT b-wave latency [(r) coef. = -0.311, -0.395, -0.362, -0.419, and -0.342].

**Conclusion:**

The retinal structure and function were significantly affected in patients with PD in comparison to healthy controls. There was a significant impact of disease duration on retinal thickness, and there was a significant negative correlation between the degree of motor dysfunction in patients with PD and retinal function.

## Introduction

Parkinson’s disease (PD) is the second most common neurodegenerative disorder affecting middle-aged and elderly people in the developed world [1]. Idiopathic PD is diagnosed based on medical history and a neurologic examination using movement Disorder Society‐Unified Parkinson's Disease Rating Scale (MDS‐UPDRS) [2]. It can take several years to get a final conclusion due to heterogeneity of PD regarding the age of onset, clinical presentation, rate of progression [3]. As a result, the diagnosis process needs to be improved and expedited in the early stages of PD using new technologies [4]. The motor symptoms of PD, including bradykinesia, rigidity, resting tremor, and postural instability, are well-known. However, dopaminergic neuronal loss can also cause non-motor changes, such as anhedonia, dementia, and autonomic dysfunction [5]. In addition, a more diffuse pathology might be associated with the non-motor symptoms as well; the cholinergic glutamatergic, noradrenergic, and serotonergic systems [6].

One of the non-motor systems affected by PD is vision. Visual acuity, contrast sensitivity, ocular motility, and color perception are among the visual abnormalities associated with PD [7]. Such vision impairment in patients with PD seems to be caused by dysfunction of the intraretinal dopaminergic circuitry and final retinal output to the brain. The human retina has dopaminergic amacrine and interplexiform cells [8]. Through dopaminergic receptors, dopamine in the mammalian retina alters colour perception and contrast sensitivity (D1 and D2) [9]. Changes in signal dispersion, colour perception, and contrast sensitivity result from a complete loss of D1 and D2 receptor activation [9].

The retina is easily accessible for clinical examination and is a component of the central nervous system. Since the majority of the retinal ganglion cells (RGCs) axons are not myelinated, the retinal nerve fibre layer (RNFL) thickness measurements offer a relatively direct evaluation of the axons and axonal damage [10].

Optical coherence tomography (OCT) is a non-invasive imaging test that uses interference patterns created by low coherence light reflected from retinal tissues to obtain cross-sectional pictures of the retina and optic disc. This approach entails the creation of parameters that offer precise, impartial, and repeatable measurements of the various retinal layers [11]. Measures of specific layers, such the RGC layer, provide more precise information regarding axonal loss in neurodegenerative illnesses, according to recent study on segmentation and analysis of several retinal layers [11].

Electrophysiological studies such as visual evoked potential (VEP) can easily detect the subtle visual impairment that may develop in PD [12]. Electroretinogram (ERG) may also provide a simple tool to evaluate retinal dopaminergic mechanisms and contribute to the clinical assessment and monitoring of dopaminergic therapy [13].

As such, the aim of this study was to evaluate the functional and structural changes in the retina in patients with PD, and to see whether these changes correlate with disease duration and degree of motor dysfunction.

## Methods

### Study design

This case–control study was conducted on patients diagnosed with idiopathic PD and age- and sex-matched healthy controls. The patients were recruited from the Neurology outpatient clinic, Beni-Suef University Hospital, during the period from January 2021 to June 2022. Healthy controls were recruited from the patients’ relatives. The study was explained to all participants and written informed consent was obtained from them or their first-degree relatives before participation in the study. The study was ethically approved by Faculty of medicine, Beni Suef University Research Ethical Committee (FM-BSU REC). The approval number is FMBSUREC/06122020/ Mohammed. All methods were carried out in accordance with relevant guidelines and regulations.

### Eligibility criteria

The study included patients fulfilling the criteria for diagnosis of Parkinson’s disease based on British Brain Bank criteria [14]. The age range was from 50–80.

Patients with the following conditions were excluded from this study: patients with secondary Parkinsonism (Drug-induced, post traumatic, or post infectious) or atypical Parkinsonism, patients having major neurocognitive disorder, patients with MRI brain showing structural lesion like multiple or extensive infarcts, severe white matter hyperintensity burden, intracerebral hemorrhage, subdural hematoma, tumors, encephalitis, or hydrocephalus, patients with significant refractive errors (> 5 diopters of spherical equivalent refraction or 3 diopters of astigmatism), intraocular pressure ≥ 21 mm Hg, media opacifications, ocular trauma, concomitant retinal or optic nerve pathology, and patient with any concomitant medical disorder known to affect the retina or optic nerve e.g. Hypertension (HTN), diabetes mellitus (DM) or autoimmune disorder.

### Measures

#### Evaluation and staging of Parkinson’s disease using

##### Modified Hoehn and Yahr staging scale (H & Y staging) [15]

It provides an overall assessment of staging of Parkinson's disease based on clinical features and functional disability. Stage 0: no signs of disease, Stage 1.0: symptoms are very mild; unilateral involvement only, stage 1.5: unilateral and axial involvement, stage 2: bilateral involvement without impaired balance, stage 2.5: mild bilateral disease with recovery on pull test, stage 3: mild to moderate bilateral disease; some postural instability; physically independent, stage 4: severe disability; still able to walk or stand unassisted, and stage 5: wheelchair bound or bedridden unless aide.

##### Unified Parkinson’s Disease Rating Scale (UPDRS) [16]

It was used as a rating scale for assessment of PD patients included in our study (on medications). It objectively rates an individual patient’s disability at a particular moment in time. Its score is a reflection of disease burden on the individual patient and is useful in describing disease progression and treatment response with time. The UPDRS is scored from a total of 195 points; higher scores reflect marked disability. It is made up of the following sections: Part I: evaluation of cognition, behavior, and mood, Part II: self-evaluation of the activities of daily life, Part III: clinician-evaluation of motor function, part IV: complication of medical treatment, and part V: other complications.

#### Optical coherence tomography (OCT)

Retinal imaging was done using RTVue-OCT ‘Optovue’ (Optovue Inc.,Fremont, California, USA) device with a software version 2018.1.1.63. The RTVue-100 is one of the SD-OCT devices with a scan rate of 26000 A scans per second and an axial resolution of 5 pm, allowing fast cross-sectional imaging of the retinal microstructure at high resolution in a rapid, objective, reproducible manner. Optic nerve head (ONH) protocol and ganglion cell complex (GCC) protocols were used. Good quality images with a signal strength index (SS1) 250 were included. OCT images were obtained from both eyes of each patient (three images were obtained and the average was taken).

##### Optic nerve head (ONH) scan

The ONH protocol was used to obtain RNFL and ONH measurements. In the measurement of RNFL parameters, ONH protocol generates a polar RNFL thickness map from which RNFL thickness is measured along a circle 3.45 mm in diameter centered on the optic disc. Parameters including overall average, superior hemisphere, inferior hemisphere, temporal quadrant, superior quadrant, inferior quadrant, and nasal quadrant were provided [17].

##### Ganglion cell complex scan (GCC)

The GCC was used to obtain macular measurements. In the measurement of macular parameters, GCC protocol scans a 7 mm square region with 15 vertical lines at 0.55 mm intervals and 1 horizontal line. Macular B-scan evaluates macular total retinal (TR) measurement in two layers: GCC and outer retina OR layers. GCC is composed of ganglion cell layer, nevre fiber layer, and inner plexiform layer. GCC parameters including overall average thickness, superior thickness, inferior thickness, superior minus inferior thickness, global loss volume (GLV), and focal loss volume (FLV) are provided [17].

The eyes of all participants were dilated using mydriatic eye drops before image acquisition. Participants were instructed to fixate on an intrinsic fixation target during the process of OCT scanning. If the participant was not fixating well and the center of image was not on the center of the fovea, a manual adjustment was performed. All OCT scans were performed by the same experienced optometrist.

#### Full-field clinical electroretinography (ffERG)

The ffERG was performed at the Neuro Diagnostic & Research Center (NDRC), Beni-Suef Hospital, Egypt, using Roland consult electrophysiological diagnostic systems [utilizing Reti-Scan 21 (Roland Consult, Brandenburg a.d. Havel, Germany)]. The parameters for the Standard flash (ganzfeld) stimuli were revised to ISCEV guidelines [18] that specified six responses based on the adaptation state of the eye and the flash strength.

1. Dark-adapted 0.01 ERG (a rod-driven response of on bipolar cells).
2. Dark-adapted 3 ERG (combined responses arising from photoreceptors and bipolar cells of both the rod and cone systems; rod dominated).
3. Dark-adapted 10 ERG (combined response with enhanced a-waves reflecting photoreceptor function).
4. Dark-adapted oscillatory potentials (responses primarily from amacrine cells).
5. Light-adapted 3 ERG (responses of the cone system; a-waves arise from cone photoreceptors and cone Off- bipolar cells; the b-wave comes from On- and Off-cone bipolar cells).
6. Light-adapted 30 Hz flicker ERG (a sensitive cone-pathway-driven response) [18].

### Statistical analysis

The data were coded and entered using: the statistical package for social science (SPSS) version 25 (Released 2017. IBM SPSS Statistics for Windows, Version 25.0. Armonk, NY: IBM Corp.). We used Kolmogorov–Smirnov test to check the normality of the quantitative variables. The data were presented using mean and standard deviation for quantitative data such as age, disease duration, H & Y staging, UPDRS, OCT, and ffERG parameters. Categorical variables such as sex and antiparkinsonian medications were presented as number and percent. Independent sample t- test was used for comparison between PD patients and controls in age, OCT, and ffERG parameters. Chi square test was for comparison between PD patients and controls in sex. The Pearson correlation coefficient (r) was used to correlate disease duration, H & Y staging, and UPDRS with OCT and ffERG parameters. The *P*-values were adjusted for multiple testing by performing the Benjamini–Hochberg procedure. *P*-value ≤ 0.05 was considered significant.

## Results

### Demographics and clinical characteristics of PD patients

This case–control study was conducted on 50 PD patients and 50 healthy controls. The mean value for age in PD patients was 60.36 ± 11.38 years, while the mean value for age in controls was 60.72 ± 11.79 years. There was no statistically significant difference between patients and controls regarding age (*P*-value = 0.77). As for sex, 68% (*n* = 34) of PD patients were males and 32% (*n* = 16) were females. As for controls, 64% (*n* = 32) were males and 36% (*n* = 18) were females. There was no statistically significant difference between patients and controls regarding sex (*P*-value = 0.673).

Regarding clinical characteristics of PD patients, the mean value for disease duration was 3.64 ± 2.32 years, for H&Y staging was 2.59 ± 0.81, and for UPDRS total score was 33.44 ± 15.12 (Table 1).

**Table 1:** Clinical characteristics of PD patients

			PD patients (*n* = 50)
Disease duration in years [mean (SD)]			3.64(2.32)
H & Y staging [mean (SD)]			2.59(0.81)
UPDRS [mean (SD)]	Motor	Tremor	5.52(3.01)
		Rigidity	5.26(2.5)
		Postural instability	0.9(0.5)
		Bradykinesia	1.24(0.55)
		Total Motor	22.04(8.45)
	Mentation		1.5(1.35)
	ADL		7.64(5.18)
	Complication		2.14(2.29)
	Total score		33.44(15.12)
Anti-parkinsonian medications	*Levodopa*		42 (84%)
	*Pramipexole*		31 (62%)
	*Amantadine*		18 (36%)
	*Biperiden*		24 (48%)
	Selegiline		12 (24%)

*ADL* Activities of daily living, *H&Y* Hoehn and Yahr, *PD* Parkinson’s disease, *UPDRS* Unified Parkinson’s Disease Rating Scale

### Reduced peripapillary RNFL thickness in PD patients

**Fig. 1:**
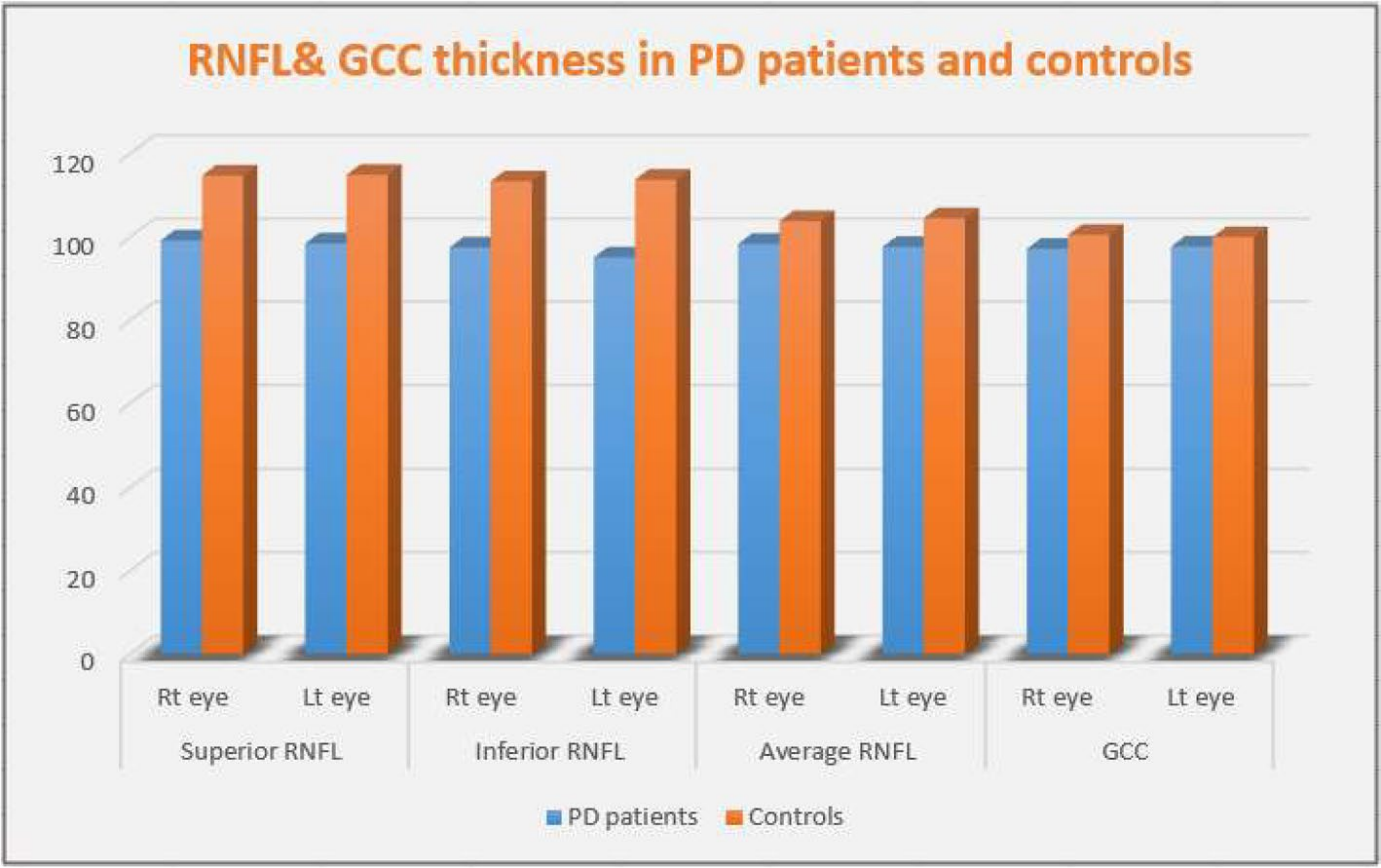
RNFL& GCC thickness in PD patients and controls GCC: ganglion cell complex, PD: Parkinson's disease, RNFL: Retinal nerve fiber layer

The peripapillary RNFL thickness (superior, inferior & average) on both sides were all significantly reduced in PD patients compared to healthy controls (*P*-value < 0.001, < 0.001, < 0.001, < 0.001, 0.003, < 0.001 respectively) (Table 2, Fig. 1).

**Table 2:** RNFL& GCC thickness in PD patients and controls

	PD patients [mean (SD)] (*n* = 50)	Controls [mean (SD)] (*n* = 50)	*P*-value
Superior RNFL			
Rt eye	99.21(11.36)	114.81(11)	< 0.001*
Lt eye	98.5(10.59)	114.99(9.83)	< 0.001*
Inferior RNFL			
Rt eye	97.53(12.21)	113.44(10.2)	< 0.001*
Lt eye	95.15(12.03)	113.83(9.96)	< 0.001*
Average RNFL			
Rt eye	98.39(10.52)	103.83(7.13)	0.003*
Lt eye	97.66(10.47)	104.56(8.2)	< 0.001*
GCC			
Rt eye	97.19(8.73)	100.57(4.97)	0.02*
Lt eye	97.76(7.79)	100.04(5.44)	0.09

The *P* values were adjusted for multiple testing using Benjamini and Hochberg procedure

*GCC* ganglion cell complex, *PD* Parkinson’s disease, *RNFL* Retinal nerve fiber layer

^*^P-value ≤ 0.05 is considered significant

The macular ganglion cell complex (GCC) thickness was significantly reduced in PD patients compared to healthy controls regarding right (RT) eye (*P*- value = 0.02), whereas, it was insignificantly reduced regarding left (LT) eye (*P*-value = 0.09) (Table 2, Fig. 1).

**Fig. 2:**
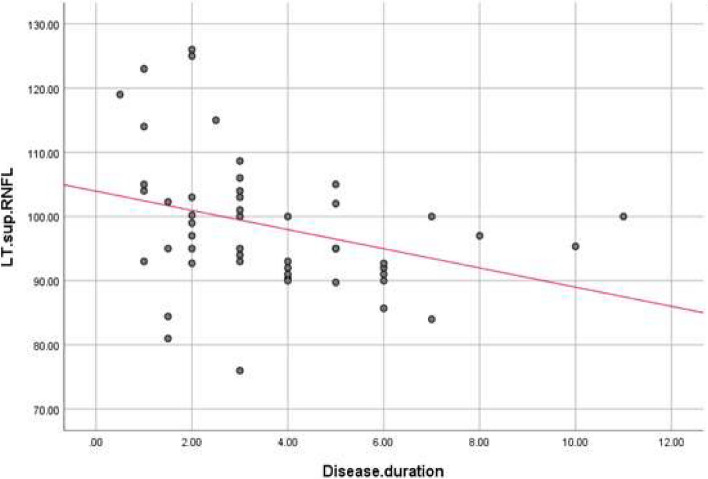
Correlations between disease duration and left superior RNFL thickness. RNFL: Retinal nerve fiber layer

**Fig. 3:**
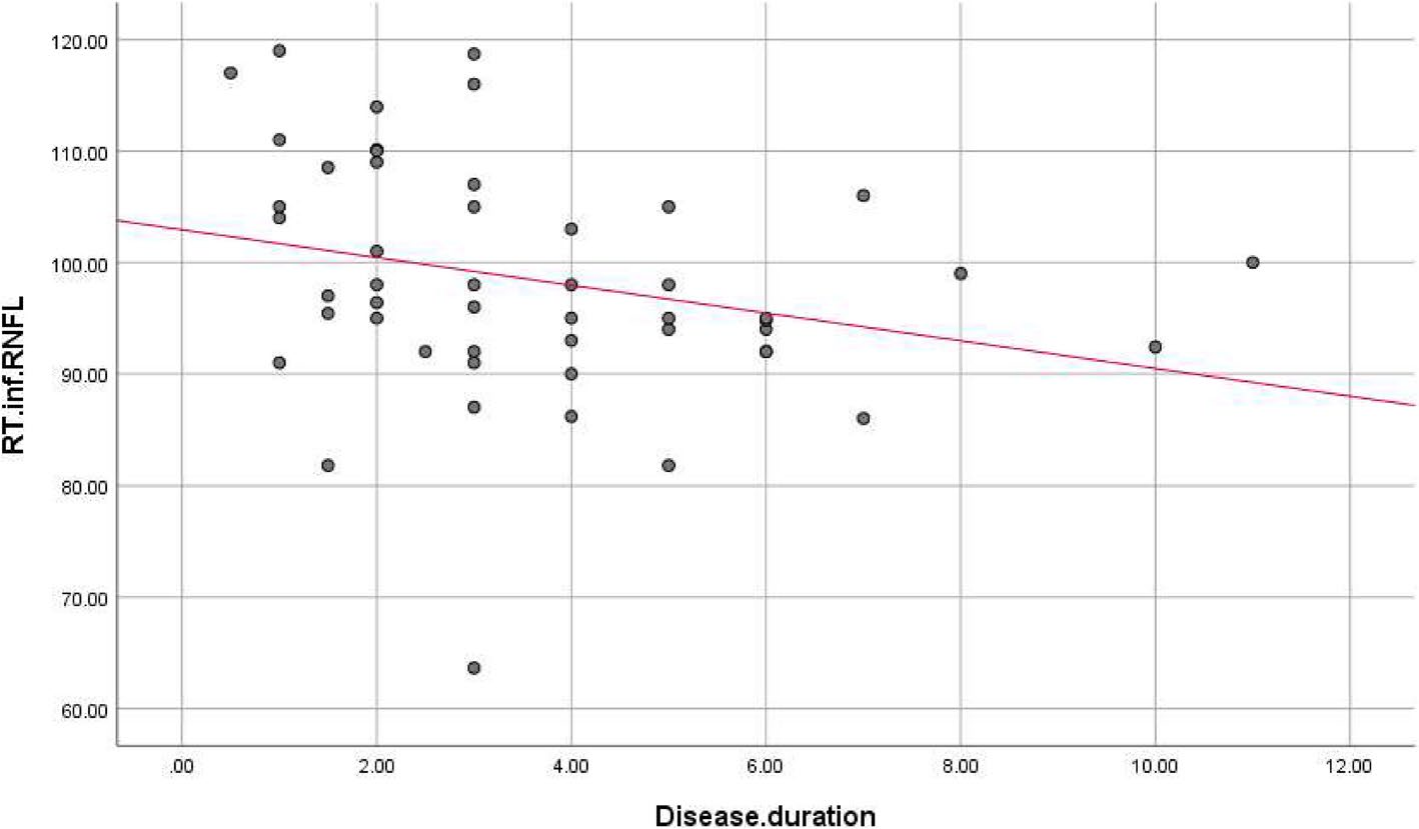
Correlations between disease duration and right inferior RNFL thickness. RNFL: Retinal nerve fiber layer

**Fig. 4:**
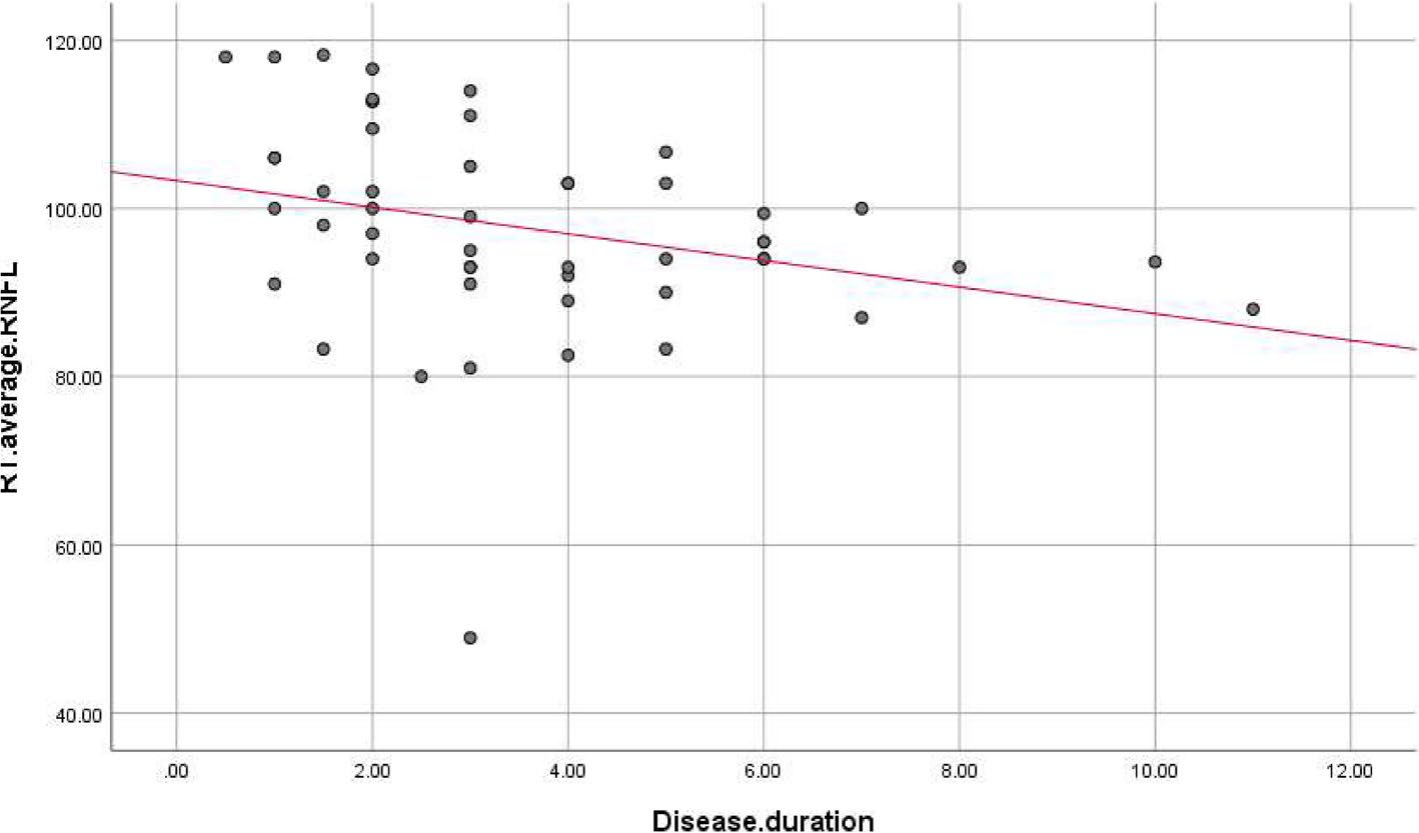
Correlations between disease duration and right average RNFL thickness. RNFL: Retinal nerve fiber layer

**Fig. 5:**
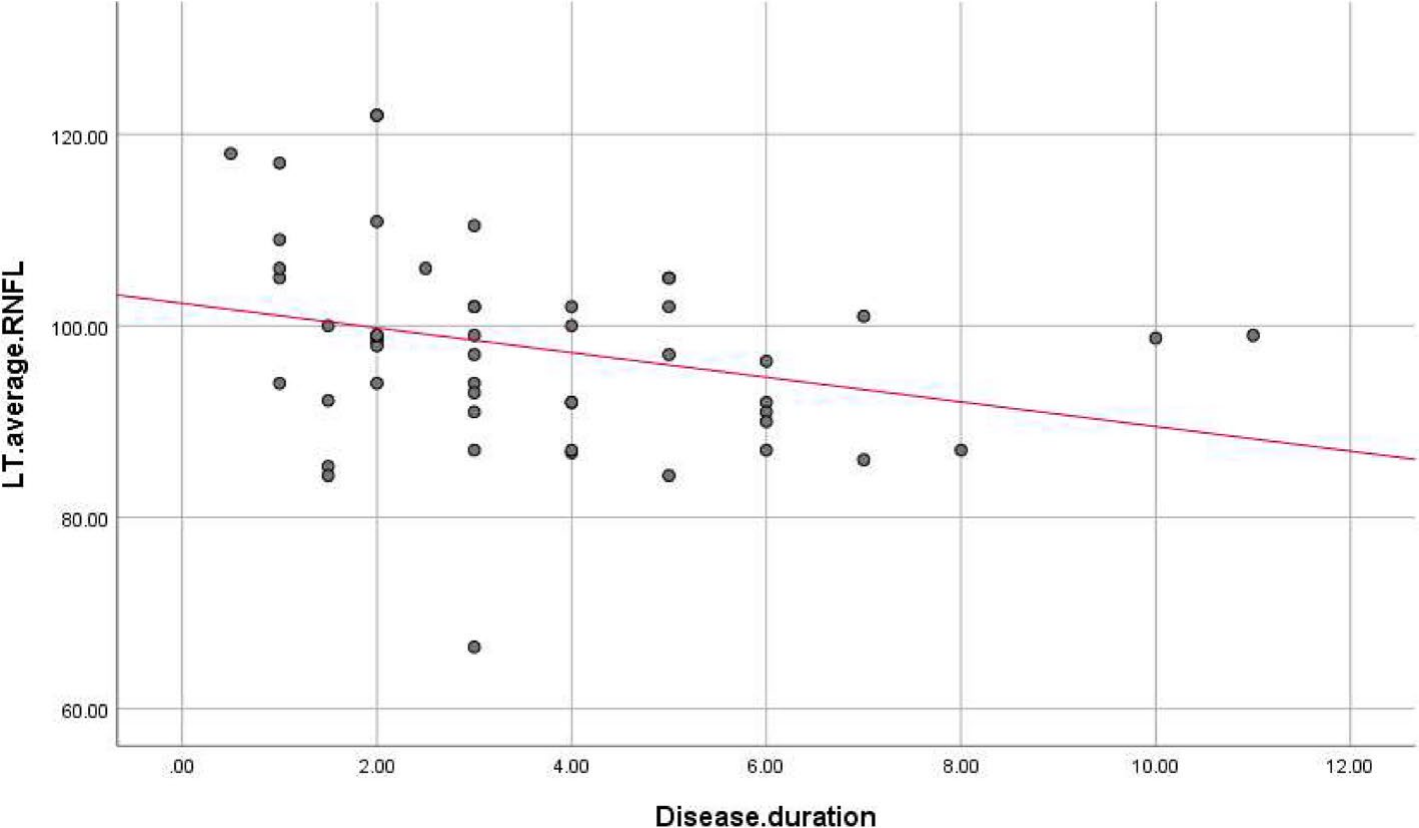
Correlations between disease duration and left average RNFL thickness. RNFL: Retinal nerve fiber layer

There were statistically significant negative correlations between disease duration, and LT superior (r. coef. = -0.327, *P*-value = 0.02), RT inferior (r. coef. = -0.301, *P*-value = 0.034), and RT average (r. coef. = -0.275, *P*-value = 0.054), and LT average RNFL thickness (r. coef. = -0.285, *P*-value = 0.045) (Table 3, Figs. 2, 3, 4 and 5).

**Table 3:** Correlation between disease duration and RNFL thickness in PD patients

	Disease duration in years	
	(r) coef	*P*-value
Superior RNFL		
Rt eye	-0.230	0.108
Lt eye	-0.327	0.02*
Inferior RNFL		
Rt eye	-0.301	0.034*
Lt eye	-0.251	0.078
Average RNFL		
Rt eye	-0.275	0.054*
Lt eye	-0.285	0.045*
Average GCC		
Rt eye	-0.262	0.066
Lt eye	-0.220	0.125

*GCC* ganglion cell complex, *RNFL* Retinal nerve fiber layer

r: Pearson Correlation Coefficient, *P-value ≤ 0.05 is considered significant

There were no statistically significant correlations between either H&Y staging or UPDRS, and OCT parameters including RNFL and GCC thickness (*P*-values > 0.05) (Table 4).

**Table 4:** Correlation between both H&Y staging and UPDRS and OCT parameters in PD patients

	H&Y staging		Total UPDRS	
	(r) coef	*P*-value	(r) coef	*P*-value
Superior RNFL				
Rt eye	-0.154	0.285	-0.131	0.364
Lt eye	-0.176	0.221	-0.180	0.211
Inferior RNFL				
Rt eye	-0.225	0.116	-0.091	0.528
Lt eye	-0.146	0.312	-0.102	0.481
Average RNFL				
Rt eye	-0.192	0.181	-0.099	0.493
Lt eye	-0.189	0.188	-0.163	0.257
Average GCC				
Rt eye	-0.132	0.362	-0.089	0.537
Lt eye	-0.152	0.292	-0.107	0.461

*GCC* ganglion cell complex, *H&Y* Hoehn and Yahr, *RNFL* Retinal nerve fiber layer, *UPDRS* Unified Parkinson’s Disease Rating Scale

r: Pearson Correlation Coefficient, *P*-value > 0.05 is considered statistically insignificant

### Delayed latency and reduced amplitude in both dark-adapted rods and the light-adapted cone for both a & b waves in PD patients

The dark-adapted rods a & b wave responses showed significantly delayed latency in PD patients as compared to healthy controls, (*P*- value < 0.001 in all comparisons). At the same time, amplitudes for a and b waves were significantly lower in PD patients as compared to healthy controls (*P*- value < 0.001 in all parameters) (Table 5).

**Table 5:** Full field Electroretinography (ff-ERG) responses in PD patients compared to healthy controls

			PD patients [mean (SD)] (*n* = 50)	Controls [mean (SD)] (*n* = 50)	*P*-value
Dark-adapted ERG (rod response)					
RT eye	a wave	Latency (milliseconds)	19.46 (3.66)	17.11(0.88)	< 0.001*
		Amplitude (µV)	35.87(7.92)	43.70(1.68)	< 0.001*
	b wave	Latency (milliseconds)	38.15(3.61)	34.33(0.92)	< 0.001*
		Amplitude (µV)	43.60(12.29)	64.60(1.85)	< 0.001*
LT eye	a wave	Latency (milliseconds)	19.75(3.94)	17.02(0.86)	< 0.001*
		Amplitude (µV)	35.46(8.09)	43.60(1.78)	< 0.001*
	b wave	Latency (milliseconds)	38.41(4.03)	34.40(0.94)	< 0.001*
		Amplitude (µV)	42.92(12.77)	65.02(2.51)	< 0.001*
Light-adapted ERG (cone response)					
RT eye	a wave	Latency (milliseconds)	18.53 ± 2.37	17.17(0.75)	< 0.001*
		Amplitude (µV)	8.36(4.82)	24.7(2.64)	< 0.001*
	b wave	Latency (milliseconds)	38.39(2.29)	33.70(1.62)	< 0.001*
		Amplitude (µV)	26.52(8.86)	51.13(6.61)	< 0.001*
LT eye	a wave	Latency (milliseconds)	18.57(2.58)	16.97(0.78)	< 0.001*
		Amplitude (µV)	8.34(4.60)	24.39(2.81)	< 0.001*
	b wave	Latency (milliseconds)	38.41(2.37)	33.78(1.57)	< 0.001*
		Amplitude (µV)	27.12(8.94)	50.76(7.24)	< 0.001*

*PD* Parkinson’s disease

^*^P-value ≤ 0.05 is considered significant

The light adapted cone a and b waves response showed significantly delayed latency in PD patients as compared to healthy controls, (*p*-values < 0.001). At the same time, amplitudes for a and b waves were significantly lower in the PD patients as compared to healthy controls (*p*-values < 0.001) (Table 5).

There were no statistically significant correlations between disease duration and either photopic or scotopic ERG parameters (*P*-values > 0.05) (Table 6).

**Table 6:** Correlation between disease duration and full field Electroretinography parameters in PD patients

			Disease duration in years	
			(r) coef	*P*-value
Light adapted ERG				
RT eye	a wave	Latency (milliseconds)	0.118	0.415
		Amplitude (µV)	0.009	0.949
	b wave	Latency (milliseconds)	0.164	0.256
		Amplitude (µV)	-0.189	0.190
LT eye	a wave	Latency (milliseconds)	0.150	0.300
		Amplitude (µV)	-0.037	0.800
	b wave	Latency (milliseconds)	0.265	0.063
		Amplitude (µV)	-0.129	0.371
Dark adapted ERG				
RT eye	a wave	Latency (milliseconds)	-0.006	0.966
		Amplitude (µV)	-0.030	0.839
	b wave	Latency (milliseconds)	0.053	0.716
		Amplitude (µV)	-0.223	0.120
LT eye	a wave	Latency (milliseconds)	0.024	0.871
		Amplitude (µV)	-0.035	0.811
	b wave	Latency (milliseconds)	0.068	0.641
		Amplitude (µV)	-0.223	0.120

*ERG* Electroretinography

r: Pearson Correlation Coefficient, *P*-value > 0.05 is considered statistically insignificant

There were no statistically significant correlations between H&Y staging and either light adapted or dark-adapted ERG parameters (*P*-values > 0.05). However, UPDRS showed a statistically significant negative correlation with the amplitude of light-adapted of both a & b wave UPDRS total score was negatively correlated with the amplitude of light-adapted of both RT and LT a & b wave [(r. coef. = -0.311, *P*-value = 0.028), (r. coef. = -0.395, *P*-value = 0.005), (r. coef. = -0.362, *P*-value = 0.011), (r. coef. = -0.419, *P*-value = 0.002) respectively] and with dark-adapted RT b-wave latency (r. coef = -0.342, *P*-values = 0.015). There were no statistically significant correlations between total UPDRS and the other ff-ERG values (*P*-values > 0.05) (Table 7).

**Table 7:** Correlation between both H&Y staging and UPDRS, and ff- ERG parameters in PD patients

			H&Y staging		Total UPDRS	
			(r) coef	*P*-value	(r) coef	*P*-value
Light-adapted ERG						
Rt eye	a wave	Latency (milliseconds)	0.037	0.801	-0.121	0.402
		Amplitude (µV)	-0.025	0.865	-0.311	0.028*
	b wave	Latency (milliseconds)	-0.059	0.684	-0.171	0.235
		Amplitude (µV)	-0.070	0.631	-0.395	0.005*
Lt eye	a wave	Latency (milliseconds)	0.032	0.826	-0.119	0.410
		Amplitude (µV)	-0.151	0.300	-0.362	0.011*
	b wave	Latency (milliseconds)	0.026	0.857	-0.068	0.637
		Amplitude (µV)	-0.115	0.427	-0.419	0.002*
Dark-adapted ERG						
Rt eye	a wave	Latency (milliseconds)	-0.062	0.673	-0.038	0.793
		Amplitude (µV)	-0.045	0.755	-0.001	0.997
	b wave	Latency (milliseconds)	-0.128	0.375	-0.342	0.015*
		Amplitude (µV)	-0.044	0.759	0.081	0.575
Lt eye	a wave	Latency (milliseconds)	-0.063	0.666	-0.009	0.953
		Amplitude (µV)	-0.049	0.735	0.004	0.980
	b wave	Latency (milliseconds)	-0.113	0.433	-0.264	0.064
		Amplitude (µV)	-0.008	0.955	0.099	0.492

*ERG* Electroretinography, *H&Y* Hoehn and Yahr, *UPDRS* Unified Parkinson’s Disease Rating Scale

r: Pearson Correlation Coefficient, *P-value ≤ 0.05 is considered significant

## Discussion

The current study revealed that the retinal structure and function were significantly affected in patients with PD in comparison to healthy controls. The retinal changes observed were significantly correlated with the disease duration and the degree of motor dysfunction.

Clearly, the superior, inferior, and average peripapillary RNFL thickness in the included PD patients in this study were significantly reduced compared to the healthy controls. These findings are in line with those of Satue et al., 2013 [19] who showed significant reduction in the inferior, inferotemporal and superotemporal RNFL thicknesses in a large sample size of 100 PD patients compared to 100 healthy controls using SD-OCT.

Moreover, Moschos MM et al., 2018 [20] observed a significant reduction in the average RNFL thickness as well as average GCC thickness in PD patients compared to controls. The same findings were also obtained by Ascaso F et al., 2013 and Pilat A et al., 2016 [21, 22]. The main explanation for these findings is fact that neurodegeneration in PD is not restricted to the brain, but also occurs in the retina [23].

On the contrary, several studies have shown non-significant differences in RNFL thickness between PD and healthy control [24, 25].

Regarding, macular GCC, the current study revealed a significant reduction in GCC thickness (in one eye but not in the other eye) in patients with PD compared to healthy controls. Other studies which used segmentation analysis, also observed a significant thinning of the GCL in patients with PD compared to healthy controls [26, 27].

The reported significant reduction in GCC in PD patients may be attributed to RNFL loss, which is suggested to produce consecutive degeneration of the RGC layer and its axons as the disease progresses [28, 29].

These conflicting results regarding the RNFL as well as GCC measurements in PD patients compared to healthy control may be attributed to difference in OCT equipment which can affect retinal measurements and to the differences in retinal segmentation algorithm [30].

With regard to disease duration, our study revealed that longer duration of the disease was associated with decreased RNFL thickness especially in LT superior, RT inferior and RT &LT average RNFL thickness. This agreed with a study conducted by Garcia-Martin et al. 2014 [31] which revealed that the inner retinal layer thicknesses was significantly thinner in PD patients with disease duration longer than 10 years compared to those with shorter disease duration. Also, El-Kattan, M.M et al., 2022 [23] reported in their study that retinal thickness inversely correlated with disease duration.

These results, therefore, reflect the presence of progressive degeneration in the retinal layers with disease progression and indicate that the neurodegenerative process runs in parallel in the brain and the retina in PD.

In contrast to our results, no abnormalities were observed in the OCT of PD patients in relation to disease duration in the study of Roth, N.M et al., 2014 [32].

In our study, we observed that there were no statistically significant correlations between the severity of motor symptoms assessed by H&Y staging and UPDRS, and OCT parameters including RNFL and GCC thickness.

Our findings were in agreement with Aydin, T.S et al., 2018 [33] who revealed non-significant correlations between structural parameters in the retina using OCT and the scores of either HY scale or UPDRS. Also**,** several studies showed the same findings [34, 35].

On the other hand, a previous study revealed that peripapillary RNFL as well as retinal and macular RNFL thicknesses were negatively correlated with H &Y [36]. Likewise, other studies reported an inverse correlation between the foveal thickness measured by TD-OCT and the UPDRS total and motor scores [37, 38]. These discrepant and equivocal data on the relationship between OCT measures and severity of motor symptoms in PD are likely to be explained by differences in the applied OCT devices and technologies.

Interestingly, Visser et al. and Lee et al. found in their studies that visual hallucinations in PD patients were associated with thinning of the inner retinal layers and, possibly, with reduced visual acuity. Lee et al. found a significant parafoveal inner nuclear layer thinning in PD patients in comparison to controls, whereas other retinal layers, including the retinal nerve fiber layer, as well as total macular thicknesses were not different. In contrast to our results, they didn’t find a significant correlation between retinal thicknesses and disease duration. The lack of correlation with disease duration was mostly attributed to the presence of early retinal involvement in PD patients due to retinal dopamine deficiency as well as deposition of abnormal alpha synuclein in the inner retinal layers. These pathological changes may cause RNFL thinning in recently diagnosed PD patients [39–41].

Regarding flash ERG, our study showed affection of both a and b waves. These were demonstrated in prolonged latencies and reduced amplitudes of the a and b waves of both dark-adapted and the light-adapted responses in PD patients compared to controls. This signifies dysfunction of both rod and cone photoreceptors as well as bipolar cells of the whole retina [42].

Similar to our findings, some studies showed significant reduction in ffERG amplitudes in PD patients relative to control groups [43–45]. Furthermore, delayed latencies of the cone or combined rod/cone ERG responses in PD patients has previously been demonstrated [41, 46, 47]. Interestingly, Barbara Nowacka et al., 2015 [46] found that ffERG changes can be detectable early in the course of the disease, even in the absence of structural retinal damage detected by OCT.

It is suggested that the reason for the susceptibility of pigment epithelial function to dopamine deficiency in Parkinson's disease may be related to its site being at the extremity away from dopamine release sites at the inner plexiform layer [41].

On the other hand, other studies have found no significant differences in ffERG latency and/or amplitude between PD patients and control [45, 48–51].

These contradictory results regarding ffERG in PD patients versus controls could be attributed to the different phenotypes, stages of PD patients and difference in disease duration, and whether the patients were on dopaminergic therapy or not.

Our study revealed that there were no statistically significant correlations between H&Y staging and either light adapted or dark-adapted ERG parameters. However, UPDRS showed statistically significant negative correlations with the amplitude of light-adapted of both a & b wave but didn’t show significant correlations with the other ffERG values.

These results come in agreement with previous studies which found that during PD, the reduced amplitudes of the photopic a and b waves were observed [43, 45].

The alterations in the retinal dopamine seem to be a primary factor coordinating shift from nighttime to daytime vision. Therefore, lower concentration of retinal dopamine in course of PD may cause disruption of the functional transition from a rod to cone dominated state [52]. This was observed as reduction in the photopic a and b-wave amplitude in the present study.

On the other hand, Mello, L. G. M. et al., 2022 [53] and Devos D et al., 2005 [49] found no statistically significant correlation between ffERG and clinical data.

The strength of our study was that it was the first study to correlate the combined structural–functional changes in the retina and optic nerve (assessed by OCT and ffERG) in patients with PD to the disease duration and the motor dysfunction. Such retinal changes may reflect the presence of dopaminergic dysfunction along the retinocortical pathway.

## Conclusion

PD patients had significantly impaired retinal structure and function in comparison to healthy controls. The retinal thickness in PD patients was negatively correlated with disease duration. The amplitude of light-adapted of both a & b wave, and the dark-adapted b-wave latency in PD patients was significantly correlated with motor function.

## Data Availability

Authors report that the datasets used and/or analyzed during the current study are available from the corresponding author on reasonable request.
